# The Prevalence of Social Withdrawal in Infants With Cleft Lip and Palate: The Feasibility of the Full and the Modified Versions of the Alarm Distress Baby Scale

**DOI:** 10.3389/fped.2022.804802

**Published:** 2022-07-07

**Authors:** Carla Pérez Martínez, Bruno Grollemund, Pascale Gavelle, Sylvie Viaux-Savelon, Antoine Guedeney

**Affiliations:** ^1^Private Practice at Psicología con bebés, Oaxaca, Mexico; ^2^Department of Dental-Facial Orthopedia, Pole of Bucco Dentaries' Medicine and Surgery, Cleft Competence Center, Strasbourg University Hospital, Strasbourg, France; ^3^Department of Maxillofacial Surgery and Plastic Hôpital Necker Enfants Malades, Paris, France; ^4^Department of Neonatology an Obstetric, University Lyon 1 and University Hospital Croix Rousse, Hospices Civils de Lyon (HCL), Lyon, France; ^5^Department of Child and Adolescent Psychiatry, Université de Paris and Bichat Claude Bernard Assistance Publique - Hôpitaux de Paris (APHP) Hôpital, Paris, France

**Keywords:** social withdrawal, ADBB scale, m-ADBB, Cleft Lip and Palate, infant development

## Abstract

**Background:**

Social withdrawal is a risk indicator for infant development with both organic and non-organic causes. Cleft lip and palate (CLP) impose a higher risk of physical and emotional distress in infants and alters parent-infant relationships. The ADBB scale is a screening tool to identify social withdrawal as a sign of distress in infants. The aim of this study is to evaluate the prevalence of social withdrawal behavior in infants with CLP using the full 8-item ADBB scale and the modified 5-item ADBB scale, and to examine the feasibility of both scales.

**Methods:**

145 infants with Cleft Lip and Palate were enrolled and video recorded during a pediatric consultation. All infants were scored by two expert raters trained in ADBB scale, and subsequently scored with the m-ADBB by an independent expert. We measured the interrater agreement for the full ADBB scale and psychometric properties of both scales.

**Results:**

The full ADBB scale identified 15.9% of infants as having social withdrawal behavior (score above cutoff ≥5). Among the infants evaluated with the m-ADBB scale, 44.9% had a score above the suggested cutoff (≥2). For both scales, the item “vocalization” showed the higher scores. We found a good internal consistency for the full ADBB (Cronbach's alpha = 0.82) and an acceptable internal consistency for the modified ADBB (Cronbach's alpha = 0.71). The interrater agreement for the full ADBB scale was excellent (kappa = 0.837). The Spearman correlation coefficient between the total scores of the two versions was 0.88 (*P* < 0.001).

**Conclusion:**

Our results indicate a relatively high prevalence of social withdrawal in infants with Cleft Lip and Palate, especially evaluated with the modified 5-item ADBB scale. We found that the full ADBB and the modified ADBB scales are feasible to use as screening tools of social withdrawal in this population.

**Clinical Trial Registration:**

This trial is registered on ClinicalTrials.gov, identifier: NCT00993993. The data is the property of *Assistance Publique, Hôpitaux de Paris*.

## Introduction

Cleft lip and palate (CLP) is the most frequent congenital craniofacial malformation in humans (1). This anomaly is immediately recognizable because of the disruptions of normal facial structure, and even though this medical condition is not a cause of mortality, it brings considerable morbidity in the affected infant and their family. Infants with CLP may endure numerous surgical and non-surgical treatments from birth until adulthood, which psychologically affects both the children and their family members.

Previous studies have found that infants with CLP may experience functional problems with feeding (swallowing and chewing), speaking (phonation), hearing and ventilation, and an increased prevalence of psychiatric illness, language disorders, Autism Spectrum Disorders (ASD), intellectual disability and behavioral problems in children with CLP (2).

Habersaat et al. (3) described that babies with CLP interact less with their mother at 2 months of age, compared with infants with no CLP. Other authors have found that babies with cleft lip showed fewer communicative signals toward their mothers, producing fewer positive vocalizations, spending less time in visual contact with their mothers, engaging less in active exploring of the environment and exhibiting more self-absorbed behavior than controls (4, 5). These behaviors are similar to the conceptualization of infant social withdrawal.

Social withdrawal behavior in infants is characterized by diminished or absence of either positive or negative behaviors, such as less frequent eye contact, fewer vocalizations or crying, a decreased level of activity, delayed reaction time, fewer facial expressions, or the presence of self-stimulation gestures (6).

Brief episodes of social withdrawal are a way that infants use to regulate the intensity of stimulation and the flow of interactions (6, 7).

In contrast, sustained social withdrawal is considered a sign of organic and emotional distress that may hinder normal development since the infant is not available for interaction with others (6).

Some of the risk factors that have been associated with social withdrawal are prematurity (8, 9), sleep and feeding disorders (10), somatic suffering (11), mother's current depressive symptoms and father's perceived moderate or poor mental health (12, 13). Interestingly, Dollberg et al. (10) observed more negative relational patterns in mother-withdrawn infant dyads in terms of higher maternal intrusiveness, lower reciprocity, and lower infant involvement.

Sustained social withdrawal may have adverse effects on infants' development, since the baby is less available for social learning (6, 14). Previous studies have found that infants' social withdrawal is associated with emotional and behavioral problems (15, 16), language development (17, 18) and cognitive milestones [e.g., (14)].

In this perspective, screening of social withdrawal could be beneficial for early detection and intervention in high-risk populations, such as infants with CLP. The ADBB scale is a brief screening tool that facilitates a more structured observation of the infant's social behavior, and that allows the detection of social withdrawal in infants aged 2–24 months (19).

The full ADBB consists of eight items: facial expression, eye contact, general level of activity, self-stimulating gestures, vocalizations, response to stimulation, relationship with the clinician, and the capacity to attract and maintain attention with the observer. Validity and reliability studies of the scale have shown good results (6). The ADBB scale demonstrated a good internal consistency for the global scale (Cronbach's alpha = 0.83), and a cutoff score of 5 was determined to be optimal for screening purposes (sensitivity of 0.82 and a specificity of 0.78) when using with French infants.

The modified version of the ADBB (m-ADBB) is a shorter instrument developed by Matthey et al. (20). In the m-ADBB, item 4 (Self-stimulating gestures) was removed because it showed poor interrater reliability; item 6 (response to stimulation) was removed because the item demonstrated high correlations with item 3 (general level of activity); and item 8 (attraction) was removed due to high correlations with six of the other items. Consequently, the m-ADBB is composed of five items from the original ADBB: Item 1 (Facial expressions), item 2 (Eye contact), item 3 (General level of activity), item 5 (Vocalizations), and item 7 (Relationship). This version is scored on a 3-point scale (0 = Satisfactory, 1 = Possible Problem, and 2 = Definite Problem). A recent study found that the m-ADBB showed better interrater reliability than the full ADBB, whereas the full ADBB reported better internal consistency than the m-ADBB (21).

In a previous study, we examined the social withdrawal in infants with CLP using the full ADBB scale (22). The aim of the present study is to evaluate the prevalence of social withdrawal behavior in infants with CLP using the full 8-item ADBB scale and the modified 5-item ADBB scale, and to examine the feasibility of both scales.

## Materials and Methods

### Participants

All infants born with a Cleft Lip (with or without cleft palate) and followed at the recruitment centers were eligible for the study and were invited to participate. The non-inclusion criteria were infants born before 37 weeks of gestation, infants whose birth weight was under 2.00 kg, placed in foster homes, whose parents showed insufficient level of French and/or were illiterate.

**Figure 1:**
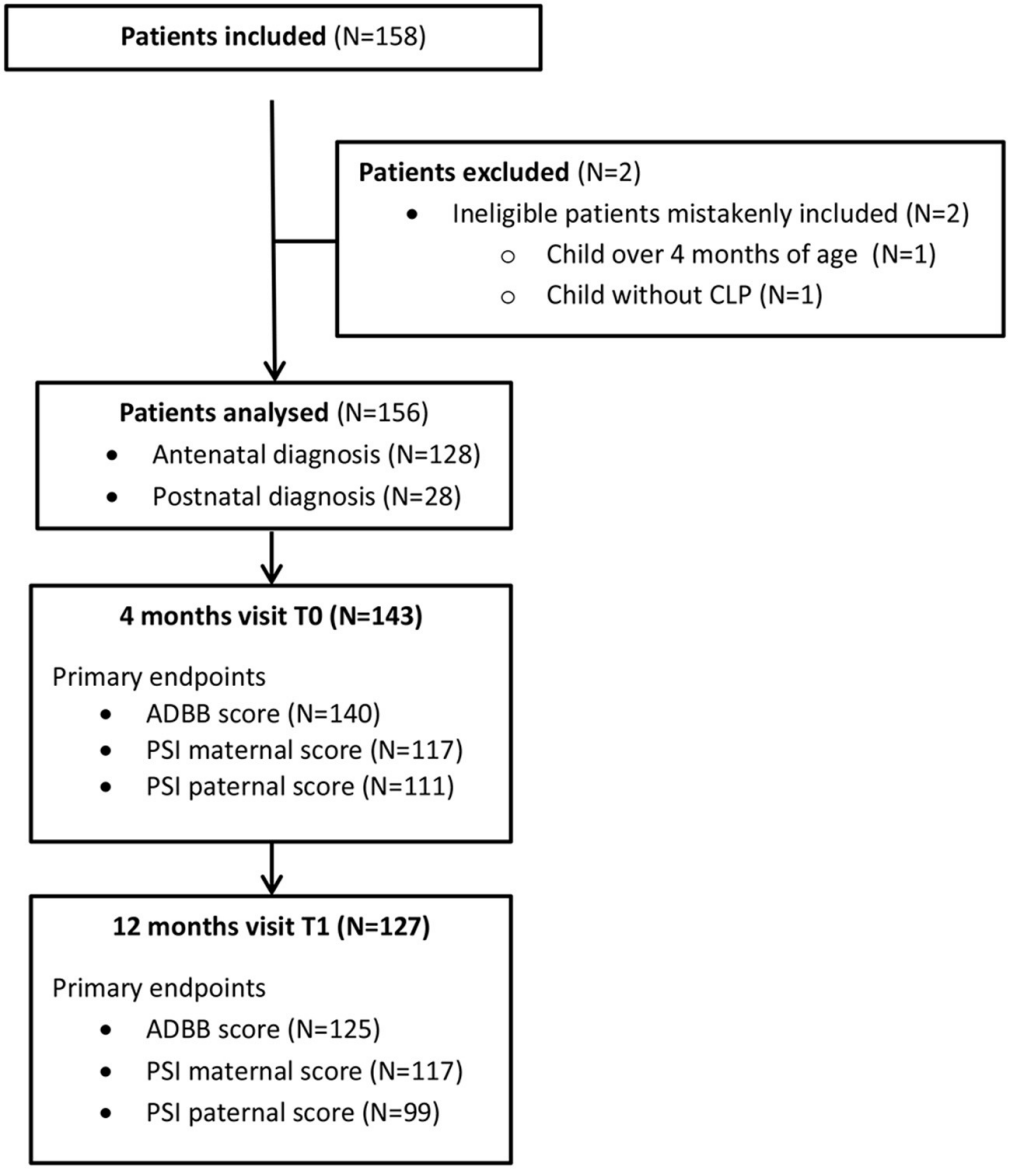
Flowchart of the study.

One hundred and forty-five infants with Cleft Lip and Palate (CLP) participated in this multi-center study in the first evaluation, and one hundred and twenty-three infants continued to participate in the second assessment. Table 1 shows the main characteristics of the participants. As we can see, the average age at the first evaluation was 4.04 months (SD = 0.65), and 12.29 months (SD = 1.24) for the second assessment. Figure 1 is the Flow chart of the study.

**Table 1:** Main descriptive statistics of the characteristics of the infants participating (*n* = 145).

		**Weeks of gestation at birth**	**Birth weight (kg.)**	**Birth height (cm.)**	**Age at first evaluation (months)**	**Age at second evaluation (months)**
*N*	Valid	99	107	93	108	93
	Missing	46	38	52	37	52
Mean		39.08	3.24	49.54	4.04	12.29
Median		39.00	3.25	50.00	4.07	12.11
Mode		38	2.64^a^	51.00	4.08	12.11
Standard deviation		1.69	0.46	2.43	0.65	1.24
Skewness		−0.591	0.035	−0.794	1.026	0.832
Std. Error skewness		0.243	0.234	0.250	0.209	0.233
Kurtosis		0.571	0.277	1.287	2.099	2.767
Std. Error Kurtosis		0.481	0.463	0.495	0.414	0.461
Minimum		34	2.15	41.00	3.07	9.00
Maximum		42	4.37	55.00	6.30	16.14
Percentile	25	38.00	3.00	48.00	11.46	48.00
	50	39.00	3.25	50.00	12.11	50.00
	75	40.50	3.52	51.00	12.90	51.00

a *There are more than one mode value. The smallest value is displayed*.

According to the type of malformation, 80.3% of the infants had a cleft lip with cleft palate (CLP), and 19.7% of infants with a cleft lip only (CL). Regarding the surgical protocols, on average, infants waited 86.79 (SD = 51.48) days between birth and the reconstruction surgery, in a range of 8–253 days. All parents signed an informed consent to their infants to be involved in the study.

### The Full Alarm Distress Baby Scale

The Alarm Distress Baby Scale (ADBB) was created to facilitate a more structured observation of social withdrawal behavior in infants from 2 to 24 months old, in the context of a routine physical exam (19). Social withdrawal behavior is assessed based on the infant's reaction to stimulation presented by the clinician in a short period of time. It is important that the clinician attempts to socially engage the infant –by talking, smiling, and touching- during examination.

The ADBB scale consists of eight items: (1) Facial expression, (2) Eye contact, (3) General level of activity, (4) Self-stimulating gestures, (5) Vocalizations, (6) Response to stimulation, (7) Relation between the infant and the clinician, and (8) Attraction, defined as the ability of the infant to attract and maintain the clinician's attention. The ADBB scale has been designed to avoid using items that show dramatic changes with development. Besides, the ADBB assessment is made within an age frame, so that the reaction of the infant is compared to what is expected at this age (23).

The rating is done immediately after observation in a live situation or on videotape by a professional trained in the scale. Each item is scored on a 5-point scale ranging from zero to four. For each item, zero represents best functioning and four, severe abnormality. Total score (range: 0–32) is calculated by summing the scores from the eight items, with higher scores indicating more social withdrawal. Studies have indicated that a total score of 5 or more is thought to be deviant and a sign of distress in the infant (19, 24–28). The full ADBB has shown good content validity as well as good psychometric properties when used with the general population (19).

### The Modified Alarm Distress Baby Scale

The m-ADBB is a shorter version of the ADBB scale that includes only five items: (1) Facial expression, (2) Eye contact, (3) General level of activity (4) Vocalizations, and (5) Relationship. Each item is rated with a three-point scale ranging from zero to two: 0 = Satisfactory, 1 = Possible problem, and 2 = Definite problem. Higher scores indicate more social withdrawal behavior. Authors suggested a cut-off score equal or greater of 2 as an indicator of social withdrawal in infants (20, 26).

In relation to its psychometric properties, Hartley et al. (29) found a good reliability of the m-ADBB (Cronbach's alpha = 0.80) in 10–12-month infants of HIV-infected mothers. Guedeney et al. (6) argued that because of its simplified coding and scoring scheme, as compared to the full ADBB, the m-ADBB may well prove to be a more practical solution for evaluating withdrawal behavior in vulnerable populations. On the other hand, Egmose et al. (30) suggested that the m-ADBB could be used as a first-line screening tool, and the full ADBB could be used to assess social withdrawal in specific at-risk populations.

### Procedure

Infants were video recorded during interaction with a certified ADBB professional in a pediatric routine exam setting, in the presence of a caregiver. During the examination, the professional tried to engage the infant in the interaction. A structured situation was developed where the examiners were at the eye level of the infant, using the same infant seat, toys and following the same routine.

Subsequently, videotapes of infant-clinical interaction were coded according to the ADBB manual (23), by an independent trained professional, then coded blindly by a second expert for the full ADBB scale. Of the 145 enrolled participants, 136 were evaluated with the modified version of the ADBB by a trained professional in the first assessment.

In the second assessment, 123 infants were evaluated with the full ADBB and the m-ADBB.

### Statistical Analyses

To determine the prevalence of social withdrawal, we described the frequencies and the percentage of the full and modified ADBB total scores. To estimate the interrater agreement for the full ADBB, we calculated the Cohen's kappa coefficient (κ) considering the scores from two certified ADBB trained professionals.

We measured the internal consistency of the full ADBB and the m-ADBB by calculating Cronbach's alpha and main descriptive statistics. Finally, Pearson correlation was used to estimate correlations between the full ADBB and the m-ADBB total scores. All statistical analyses were performed with the Statistical Package for the Social Sciences (SPSS).

## Results

### Prevalence of Social Withdrawal

**Figure 2:**
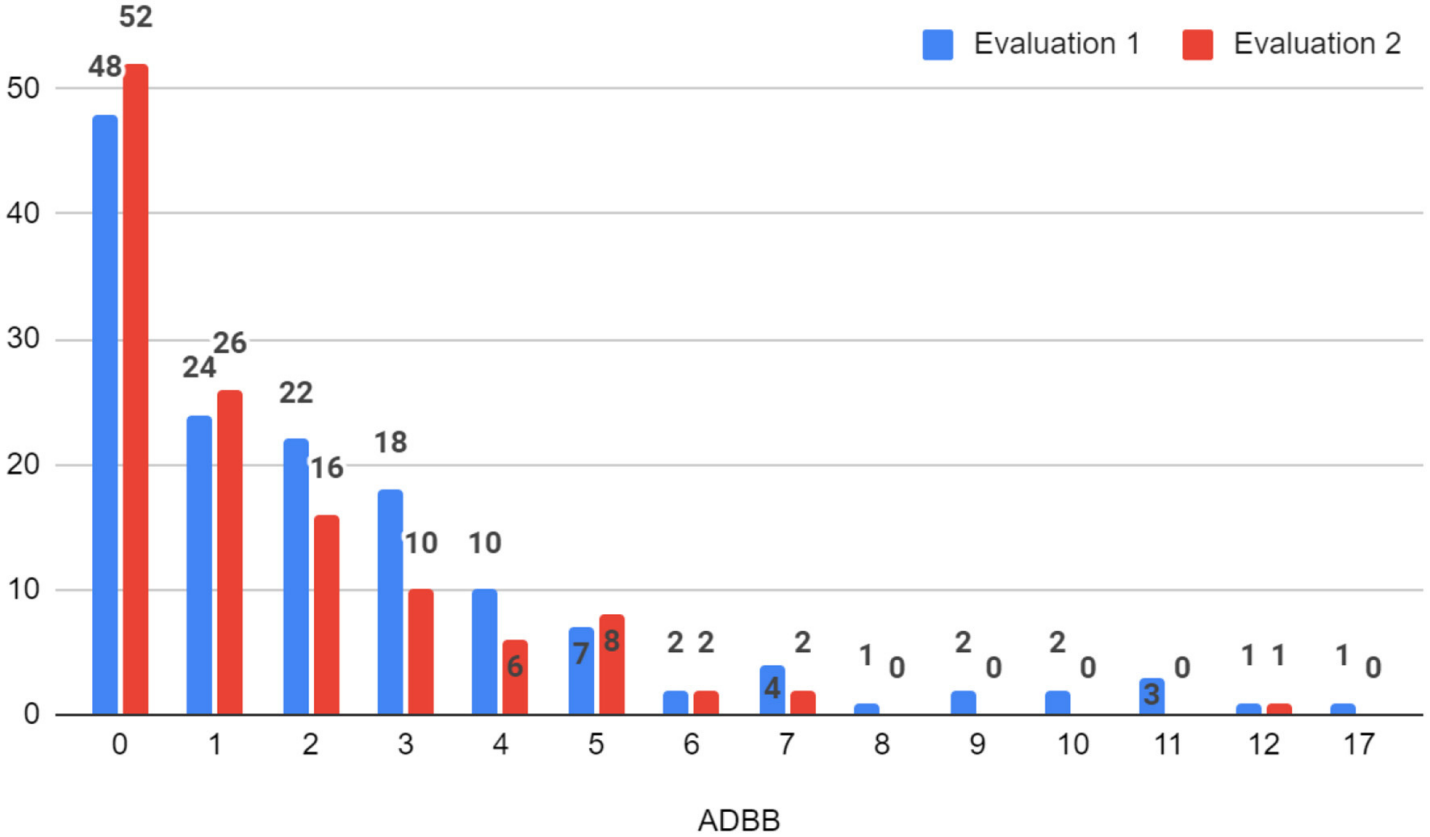
Frequencies of the total scores of the ADBB scale.

According to the full ADBB scale, the prevalence of social withdrawal (i.e., total score ≥ 5) in the first evaluation was 15.9% (23 infants), of which 5 infants had scores above 10 that suggests severe withdrawal (see Figure 2). In the second evaluation, 10.6% (13) of the infants were socially withdrawn, only 1 infant reported a score corresponding to severe withdrawal behavior (>10) (see Figure 2).

**Figure 3:**
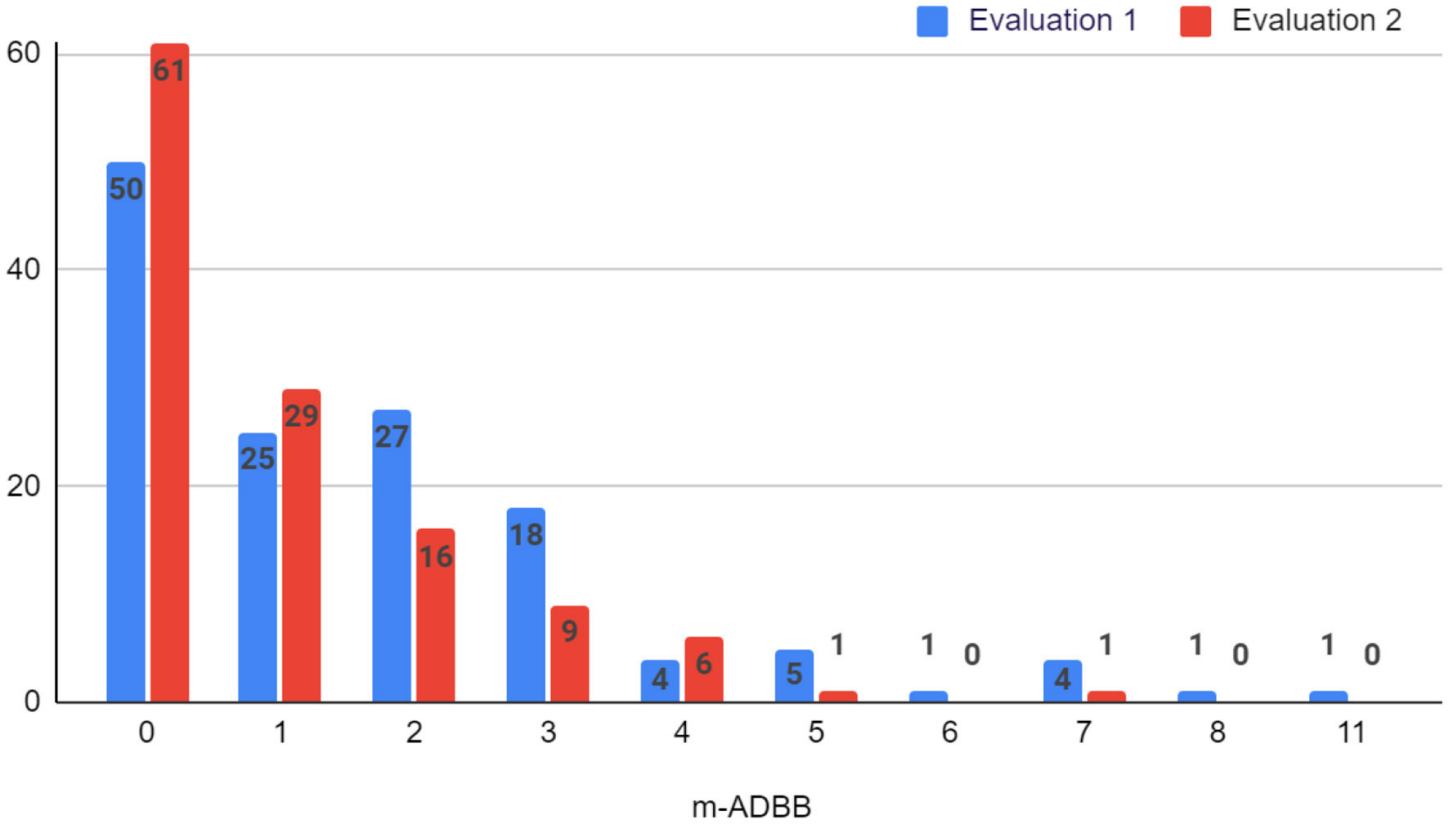
Frequencies of the total scores of the m-ADBB scale.

Using the m-ADBB, we found a social withdrawal prevalence of 44.9% (61 infants) in the first evaluation (i.e., total score ≥ 2) and 26.8% (33 infants) in the second assessment (see Figure 3).

### Internal Consistency of the Full ADBB

In the first assessment, the full ADBB scale showed a good internal consistency (Cronbach's alpha = 0.82, 95% IC 0.77 to 0.86; *n* = 145), and an excellent interrater agreement (kappa = 0.837).

The values of the correlation coefficients between the items and the total score fluctuated from 0.38 to 0.69. Item 1 “facial expression” and item 8 “attraction” showed the strongest correlations. The analysis of internal consistency indicated that the elimination of any of the items did not improve Cronbach's alpha value (see Table 2).

**Table 2:** Internal consistency of the ADBB scale in the first evaluation.

**Items**	**Mean**	**SD**	**Corrected item-total correlation**	**Cronbach's alpha if item deleted**
1. Facial expression	0.39	0.63	0.697	0.774
2. Eye contact	0.21	0.51	0.548	0.798
3. **General level of activity**	0.16	0.39	0.425	0.814
4. Self-stimulating gestures	0.17	0.49	0.388	0.817
5. **Vocalizations**	0.45	0.68	0.428	0.819
6. Response to stimulation	0.28	0.51	0.569	0.795
7. Relationship	0.33	0.58	0.590	0.791
8. Attraction	0.39	0.65	0.695	0.774

In the second assessment, the ADBB scale showed an acceptable Cronbach's alpha coefficient of 0.73 (95% IC 0.65 to 0.79; *n* = 123), and a good inter-rater agreement (kappa = 0.879). Table 3 shows the values of the correlation coefficients between the items and the total score (from 0.23 to 0.68). Item 1 “facial expression” showed the strongest correlation to the total score, and item 5 “vocalizations” reported the lowest correlation. The analysis of internal consistency indicated that the elimination of item 5 would improve Cronbach's alpha value.

**Table 3:** Internal consistency of the ADBB scale in the second evaluation.

**Items**	**Mean**	**SD**	**Corrected item-total correlation**	**Cronbach's alpha if item deleted**
1. Facial expression	0.29	0.49	0.688	0.642
2. Eye contact	0.13	0.38	0.465	0.698
3. **General level of activity**	0.07	0.25	0.514	0.704
4. Self-stimulating gestures	0.08	0.40	0.364	0.716
5. **Vocalizations**	0.38	0.58	0.239	0.761
6. Response to stimulation	0.12	0.33	0.326	0.722
7. Relationship	0.21	0.43	0.417	0.706
8. Attraction	0.26	0.48	0.551	0.677

According to the full ADBB, infants reported the highest mean on item 5 “vocalizations”, and the lowest mean on item 3 “general level of activity” in the first and the second assessments. These results suggested that infants with CLP showed more difficulties in the oral expression (less positive and negative vocalizations), and they performed better at the level of corporal activity in both assessments.

### Internal Consistency of the m-ADBB

In the first evaluation, the m-ADBB scale reported a Cronbach's alpha coefficient of 0.71 (95% IC 0.64 to 0.79; *n* = 136), which is an acceptable internal consistency. The values of the correlation coefficients between the item and the total score reached from 0.38 to 0.63. Item 1 “facial expression” showed the strongest correlation to the total score. The analysis of internal consistency indicated that the elimination of any of the items did not improve Cronbach's alpha value (see Table 4).

**Table 4:** Internal consistency of the m-ADBB scale in the first evaluation.

**Items**	**Mean**	**SD**	**Corrected item-total correlation**	**Cronbach's alpha if item deleted**
1. Facial expression	0.44	0.64	0.636	0.597
2. Eye contact	0.18	0.49	0.492	0.668
3. General level of activity	0.18	0.41	0.389	0.705
4. **Vocalizations**	0.46	0.69	0.393	0.716
5. Relationship	0.40	0.61	0.525	0.650

In the second evaluation, the m-ADBB showed a Cronbach's alpha coefficient of 0.65 (95% IC 0.54 to 0.74; *n* = 123). In line with the full ADBB results, we found that item 1 “facial expression” showed the strongest correlation to the total score, and item 5 “vocalizations” reported the lowest correlation. The analysis of internal consistency indicated that the elimination of item 5 would improve Cronbach's alpha value (see Table 5).

**Table 5:** Internal consistency of the m-ADBB scale in the second evaluation.

**Items**	**Mean**	**SD**	**Corrected item-total correlation**	**Cronbach's alpha if item deleted**
1. Facial expression	0.28	0.49	0.650	0.451
2. Eye contact	0.11	0.34	0.344	0.624
3. General level of activity	0.07	0.25	0.411	0.616
4. **Vocalizations**	0.36	0.56	0.282	0.690
5. Relationship	0.20	0.40	0.461	0.571

Regarding the m-ADBB, findings reflected a pattern like that observed in the full ADBB, where item 5 “vocalizations” showed the highest mean, and item 3 “general level of activity” reported the lowest means in the first and the second evaluations.

### Correlation Between the Full ADBB and the m-ADBB

To evaluate the possible association between the full ADBB and m-ADBB total scores, a Pearson's correlation was carried out. A statistically significant, strong, and proportional linear association was found between the full ADBB and m-ADBB total scores in the first (*r* = 0.88, *p* < 0.001, CI 95% 0.91, 0.84), and in the second assessments (*r* = 0.90, *p* < 0.001, CI 95% 0.93, 0.85). These results suggested that when the full ADBB total score increases, also the value of m-ADBB total score rises, and vice versa.

## Discussion

This is the first study evaluating the prevalence of social withdrawal using both the full and the modified version of the ADBB in infants with Cleft Lip and Palate (CLP) and examining the internal consistency of both scales.

When using the full ADBB, a prevalence of 15.9 and 10.6% of social withdrawal behavior in infants with CLP was found in the first and second evaluations, respectively. These percentages are similar to the 13% reported by Guedeney et al. (31), and the 14% observed by Guedeney et al. (2012), in infants belonging to at-risk population.

According to the modified ADBB scale, the prevalence of infant social withdrawal was 44.9% (61 infants) in the first evaluation, and 26.8% (33 infants) in the second assessment. These results are similar to the 45.5% of social withdrawal found in infants with a congenital heart disease observed by Re et al. (32). Likewise, in a study with 83 mother-infant HIV infected dyads, Durandt (33) suggested a 31% of social withdrawal behavior, with the m-ADBB using a cutoff of 2. In another study, Tauber et al. (34) reported a 62% of social withdrawal in infants –under 6 months- with a genetic diagnosis of Prader-Willi Syndrome. In this line, a 61.8% of premature infants reported signs of social withdrawal behavior, and this behavior was greater in infants who required admission to the Neonatal Intensive Care Unit or oxygen support (35). These findings emphasize the necessity of the detection of social withdrawal behavior, as a silent signal of suffering, especially in infants with a medical condition.

The internal consistency of the full ADBB in both evaluations was good and in line with the one found in the original ADBB scale used in the general population (19). In the second evaluation, the analysis of internal consistency suggested that the elimination of item 5 would improve Cronbach's alpha value, however, this item provides important information about infant's development, especially in the case of CLP patients. In both assessments, we obtained an excellent interrater agreement for the full ADBB scale, demonstrating consistency of the scorings.

Regarding the m-ADBB, we found a lower internal consistency, compared to the full ADBB, which might be due to the lower number of items (five items compared to eight). Similar results were observed by Ulak et al. (21) in a Nepali study using the GLB coefficient. They reported a 0.74 and 0.46 GLB coefficients, suggesting a good internal consistency for the full ADBB and a poor internal consistency for the m-ADBB. In contrast, Egmose et al. (30) observed that the validity of the full ADBB might be improved with the removal of items 4, 6, and 8 as suggested in the m-ADBB, using Item Response Theory. We did not obtain the interrater agreement for the m-ADBB, however, previous studies have shown promising interrater agreement using the m-ADBB (6, 21, 33), since in the modified version, the items that were difficult to score and highly correlated with each other were removed (20).

The difference in the prevalence of social withdrawal between the full and the modified versions might be explained because the m-ADBB has a poorer accuracy compared with the full ADBB. In another study using the full ADBB and the m-ADBB in the same sample, a similar pattern was found: the prevalence of social withdrawal increased when evaluating with the m-ADBB (21). Authors explained that this difference could be due to the m-ADBB being a cruder measure. More studies using the full and the modified ADBB versions in the same sample are warranted to better understand this difference.

Using a cut—off score at 3 and over for the m-ADBB would yield a prevalence of 25% for the first assessment at 4 months of age with 34 infants, and 13.82% with 17 infants at the second assessment at 1 year of age, while a cut off score of 4 and over. A cut-off score of 4 and over gives 11.76% (16 infants) at the first assessment and 6.5% at the second one (eight infants). Therefore, the cut off score of 4 with the m-ADBB seems the best to use on infants with cleft and lip palate cleft. When using the full ADBB, a prevalence of 15.9 and 10.6% of social withdrawal behavior in infants with CLP was found in the first and second evaluations, respectively.

In both evaluations, using the full ADBB and the m-ADBB infants has shown higher difficulties on the item 5 “vocalizations”. This result suggested that infants with CLP had less vocalizations expressing pleasure (i.e., cooing, laughing, or babbling) but also showing displeasure or pain (i.e., squealing, screaming, or crying). These findings might be explained if we consider that Cleft Lip/Palate malformation affects the oral cavity. The oral structural deficit itself, the post-surgery discomfort, and the use of oral devices–such as palatal obturators- to modify oral structures might complicate the production of vocalizations.

Our results are consistent with previous studies (4, 5) suggesting that infants with CLP make fewer communicative signals, and produce fewer positive vocalizations, in comparison with infants with no CLP. Likewise, Chapman et al. (36) found that babies with cleft palate had smaller canonical babbling ratios than their age-matched peers, with just 57% of the babies with cleft palate reaching the canonical babbling stage by 9 months compared to 93% of the non-cleft babies. This might explain the findings in our study, in which item 5 “vocalizations” was the dimension with major difficulties.

Finally, the strong and statistically significant correlation between the total score of the full and the modified versions demonstrates that when the total score of the full ADBB increases, also the modified version does. Ulak et al. (21) also reported highly significant correlations between the total scores of the two versions (full ADBB and m-ADBB).

Some strengths of this study are the large sample size of infants with CLP included, and the use of the ADBB and m-ADBB simultaneously, as objective screening tools. All the ADBB raters were certified trained professionals with experience in infant development. One limitation of this study is that only 136 of the 145 infants were evaluated with the m-ADBB in the first evaluation, and that only one expert scored with the m-ADBB, so we did not have reference scores to evaluate the interrater agreement. Fifteen percent of the sample was missing in the second evaluation which potentially leads to a bias in this sample. The discontinuation of participation was due to different reasons: moving out of the city, loss of contact and refusal to continue to participate mainly attributable to the distance between home and the healthcare center, and the repeated visits required for the infant's care (37).

## Conclusion

The results of this study suggest that the full and the modified versions of the ADBB are feasible to use as screening instruments of social withdrawal in infants with Cleft Lip and Palate. Both scales reported good and acceptable internal consistency, respectively. More studies are needed to further explore the psychometric characteristics of the modified ADBB scale in different populations and to examine the criterion-related validity of the full and the m-ADBB. The prevalence of social withdrawal in this population was higher than in the general population, which highlights the importance of using screening tools in primary care that evaluate the socio-emotional development in infants with CLP since the first months of life. The use of the ADBB scale facilitates a more comprehensive perception of the infants with CLP, promoting an early and individualized intervention, coordinated by the psychologist or psychiatrist that supports both the infant and their parents.

## Data Availability Statement

The raw data supporting the conclusions of this article will be made available by the authors, without undue reservation.

## Ethics Statement

The studies involving human participants were reviewed and approved by the Comité de Protection des Personnes Est IV of the Teaching Hospital from Strasbourg approved this research project on 11/18/2009, for the four study sites. Written informed consent to participate in this study was provided by the participants' legal guardian/next of kin.

## Author Contributions

This project was designed and led by BG and AG. PG led the infant and parents' clinical evaluations. AG trained CP and the several teams involved. CP carried out independent and blind ratings of all available video clips of the ADBB and m-ADBB assessments. SV-S helped in rewriting and editing of the paper. All authors have read and approved the final manuscript.

## Funding

This study was supported by the French Ministry of Health N°IDRCB: 2009-A00640-57.

## Conflict of Interest

The authors declare that the research was conducted in the absence of any commercial or financial relationships that could be construed as a potential conflict of interest.

## Publisher's Note

All claims expressed in this article are solely those of the authors and do not necessarily represent those of their affiliated organizations, or those of the publisher, the editors and the reviewers. Any product that may be evaluated in this article, or claim that may be made by its manufacturer, is not guaranteed or endorsed by the publisher.

## References

[1] Martelli D Coletta R Oliveira EA Swerts MSO Rodrigues LAM Oliveira MC. Association between maternal smoking, gender, and cleft lip and palate. Braz J Otorhinolaryngol. (2015) 81:514–9. 10.1016/j.bjorl.2015.07.011 26277833 PMC9449023

[2] Tillman KK Hakelius M Höijer J Ramklint M Ekselius L Nowinski D. Increased risk for neurodevelopmental disorders in children with orofacial clefts. J Am Acad Adolesc Psychiatry. (2018) 57:876–83. 10.1016/j.jaac.2018.06.024 30392629

[3] Habersaat S Monnier M Peter C Bolomey L Borghini A Despars J. Early mother-child interaction and later quality of attachment in infants with orofacial cleft compared to infants without cleft. Cleft Palate Craniofac J. (2013) 50:704–12. 10.1597/12-094.1 24218985

[4] Montirosso R Fedeli C Murray L Morandi F Brusati R Perego G. The role of negative maternal affective states and infant temperament in early interactions between infants with cleft lip and their mothers. J Pediatr Psychol. (2012) 37:241–50. 10.1093/jpepsy/jsr089 22004886

[5] Murray L Hentges F Hill J Karpf J Mistry B Kreutz M. The effect of cleft lip and palate, and the timing of lip repair on mother-infant interactions and infant development. J Child Psychol Psychiatry. (2008) 49:115–23. 10.1111/j.1469-7610.2007.01833.x 17979962

[6] Guedeney A Matthey S Puura K. Social withdrawal behavior in infancy: a history of the concept and a review of published studies using the alarm distress baby scale. Infant Ment Health J. (2013) 34:516–31. 10.1002/imhj.21412

[7] Brazelton BT Kolowski B Main M. The origins of reciprocity. In: Lewis M Rosenblum LD editors. The Effect of the Infant on its Caregiver Wiley Interscience. New York, NY: Wiley (1974). p. 137−54.

[8] Braarud HC Slinning K Moe V Smith L Vannebo UT Guedeney A. Relation between social withdrawal symptoms in full term and premature infants and depressive symptoms in mothers: a longitudinal study. Infant Ment Health J. (2013) 34:532–41. 10.1002/imhj.2141427429050

[9] Moe V Braarud H Wentzel-Larsen T Slinning K Vannebo UT Guedeney A. Precursor of social emotional functioning among full-term and preterm infants at 12 months: early infant withdrawal behavior and symptoms of maternal depression. Infant Behav Dev. (2016) 44:159–68. 10.1016/j.infbeh.2016.06.012 27429050

[10] Dollberg D Feldman R Keren M Guedeney A. Sustained withdrawal behavior in clinic referred and non-referred infants. Infant Ment Health J. (2006) 27:292–309. 10.1002/imhj.20093 28640475

[11] Puura K Mäntymaa M Luoma I Kaukonen P Guedeney A Salmelin R. Infant's social withdrawal symptoms assessed with a direct method in primary health care. Infant Behav Dev. (2010) 33:579–88. 10.1016/j.infbeh.2010.07.009 20723997

[12] Mäntymaa M Puura K Luoma I Kaukonen P Salmelin RK Tamminen T. Infants' social withdrawal and parents' mental health. Infant Behav Dev. (2008) 31:606–13. 10.1016/j.infbeh.2008.07.005 18774609

[13] Puura K Mäntymaa M Leppänen J Peltola M Salmellin R Luoma I. Associations between maternal interaction behavior, maternal perception of infant temperament, and infant social withdrawal. Infant Ment Health J. (2013) 34:586–93. 10.1002/imhj.21417

[14] Smith-Nielsen J Lange T Wendelboe KI Wowern RK Væver MS. Associations between maternal postpartum depression, infant social behavior with a stranger, and infant cognitive development. Infancy. (2019) 24:663–70. 10.1111/infa.12287 32677250

[15] Guedeney A Pingault J-B Thorr A Larroque B. Social withdrawal at 1 year is associated with emotional and behavioural problems at 3 and 5 years: the Eden mother-child cohort study. Eur Child Adolesc Psychiatry. (2014) 23:1181–8. 10.1007/s00787-013-0513-8 24464247

[16] Zhou F Huang P Wei X Guo Y Lu J Feng L. Prevalence and characteristics of social withdrawal tendency among 3–24 months in China: a pilot study. Front Psychiatry. (2021) 12:537411. 10.3389/fpsyt.2021.537411 34220558 PMC8242944

[17] Guedeney A Forhan A Larroque B Agostini M Pingault JB Heude B. Social withdrawal behavior at one year of age is associated with delays in reaching language milestones in the EDEN mother-child cohort study. PLoS ONE. (2016) 11:0158426. 10.1371/journal.pone.0158426 27391482 PMC4938506

[18] Milne L Greenway P Guedeney A Larroque B. Long term developmental impact of social withdrawal in infants. Infant Behav Develop. (2009) 32:159–66. 10.1016/j.infbeh.2008.12.006 19185351

[19] Guedeney A Fermanian J. A validity and reliability study of assessment and screening for sustained withdrawal reaction in infancy: the alarm distress baby scale. Infant Ment Health J. (2001) 22:559–75. 10.1002/imhj.1018

[20] Matthey S Crncec R Hales A Guedeney A. A description of the Modified Alarm Distress Baby Scale (m-ADBB): an instrument to assess for infant social withdrawal. Infant Ment Health J. (2013) 34:602–9. 10.1002/imhj.21407

[21] Ulak M Ranjitkar S Shrestha M Braarud HC Chandyo RK Shrestha L. The feasibility of the full and modified versions of the Alarm Distress Baby Scale (ADBB) and the prevalence of social withdrawal in infants in Nepal. Front Psychol. (2020) 11:2025. 10.3389/fpsyg.2020.02025 32982842 PMC7479187

[22] Grollemund B Dissaux C Gavelle P Pérez-Martínez C Mullaert J Alfaiate T. The impact of having a baby with cleft lip and palate on parents and on parent-baby relationship: the first French prospective multicentre study. BMC Pediatr. (2020) 20:230. 10.1186/s12887-020-02118-5 32423402 PMC7236125

[23] Guedeney A. Alarm Distress Baby Scale: A Recent Manual for Use. Paris (2015).

[24] De Rosa E Curró V Wendland J Maulucci S Maulucci ML De Giovanni L. Propriétés psychométriques de l'échelle Alarme Détresse Bébé (ADBB) appliquée à 81 enfants italiens. Dépression postnatale, maladie somatique maternelle et retrait chez l'enfant. Devenir. (2010) 3:209–23. 10.3917/dev.103.0209

[25] Facuri-Lopes S Ricas J Cotta-Mancini M. Evaluation of the psychometric properties of the Alarm Distress Baby Scale among 122 Brazilian children. Infant Ment Health J. (2008) 29:153–73. 10.1002/imhj.20169 28636194

[26] Matthey S Guedeney A Starakis N Barnett B. Assessing the social behavior of infants: use of the ADBB scale and relationship to mother's mood. Infant Ment Health J. (2005) 26:442–58. 10.1002/imhj.20061 28682495

[27] Oliver M Yulitta H Guedeney A. Análisis psicométrico de la Escala de alarma sobre el retraimiento del bebé de uso pediátrico. Acta Psiquiátr Psicol Am Latina. (2016) 62:77–84.

[28] Puura K Guedeney A Mäntymaa M Tamminnen T. Detecting infants in need: are complicated measures really necessary? Infant Ment Health J. (2007) 28:409–21. 10.1002/imhj.20144 28640405

[29] Hartley C Pretorius K Mohamed A Laughton B Madhi S Cotton MF. Maternal postpartum depression and infant social withdrawal among human immunodeficiency virus (HIV) positive mother–infant dyads. Psychol Health Med. (2010) 15:278–87. 10.1080/13548501003615258 20480433

[30] Egmose E Smith-Nielsen J Lange T Stougaard M Stuart A Guedeney A. How to screen for social withdrawal in primary care: an evaluation of the alarm distress baby scale using item response theory. Int J Nurs Stud Adv. (2021) 3:100038. 10.1016/j.ijnsa.2021.100038 PMC11080292 38746716

[31] Guedeney A Foucault C Bougen E Larroque B Mentré F. Screening for risk factors of relational withdrawal behavior in infants aged 14–18 months. Eur Psychiatry. (2008) 23:150–5. 10.1016/j.eurpsy.2007.07.008 17904336

[32] Re J Dean S Mullaert J Guedeney A Menahem S. Maternal distress and infant social withdrawal (ADBB) following infant cardiac surgery for congenital heart disease. World J Pediatr Congenit Heart Surg. (2018) 9:624–37. 10.1177/2150135118788788 30322360

[33] Durandt ND. Outcome of Home-Visiting Intervention to Improve Social Withdrawal Assessed with the m-ADBB in Six-Month Old Infants in Khayelisha, Cape Town: A Cluster Randomised Controlled Trial (thesis). Stellenbosch University. Available online at: https://scholar.sun.ac.za/handle/10019.1/96009 (accessed September 8, 2021).

[34] Tauber M Boulanouar K Diene G Çabal-Berthoumieu S Ehlinger V Fichaux-Bourin P. The use of oxytocin to improve feeding and social skills in infants with Prader–Willi syndrome. Pediatrics. (2017) 139:e20162976. 10.1542/peds.2016-2976 28100688

[35] Pérez-Martínez C Oliver M Fernández S Viaux-Savelon S. Análisis del retraimiento social infantil en participantes en el Programa Madre Canguro. Acta Psiquiátr Psicol Amé Latina. (2020) 66:188–96.

[36] Chapman KL Hardin-Jones M Schulte J Halter K. Vocal development of 9-month-old babies with cleft palate. J Speech Lang Hear Res. (2001) 44:1268–83. 10.1044/1092-4388(2001/099) 11776364

[37] Grollemund B Guedeney A Vázquez MP Picard A Soupre V Pellerin P. Relational development in children with cleft lip and palate: influence of the waiting period prior to the first surgical intervention and parental psychological perceptions of the abnormality. BMC Pediatr. (2012) 12:65. 10.1186/1471-2431-12-65 22682069 PMC3436730

